# The effects of socioeconomic status on perceived self‐efficacy among caregivers of persons with Alzheimer's disease and related dementias

**DOI:** 10.1002/alz70858_105274

**Published:** 2025-12-26

**Authors:** Miriam J. Rodriguez, Jordan R. Hill, Bailey Gardner, Mariana D. Stavig, Lilian Golzarri‐Arroyo, Anna L. M. Macagno, Roger S. Zoh, Richard Holden, Malaz Boustani

**Affiliations:** ^1^ Indiana University School of Public Health, Bloomington, IN, USA; ^2^ Indiana Clinical Translational Science Institute, Indianapolis, IN, USA; ^3^ Regenstrief Institute, Indianapolis, IN, USA; ^4^ Indiana University School of Medicine, Indianapolis, IN, USA

## Abstract

**Background:**

Among ADRD caregivers, self‐efficacy, defined as a “person's belief about their ability to organize and execute courses of action to manage given situations,” is associated with reduced caregiver burden. Socioeconomic status (SES) has been associated with health outcomes, but little is known about how it impacts self‐efficacy in this population. The current aim is to study this association across three self‐efficacy domains: 1) obtaining respite, 2) responding to disruptive patient behaviors (RDPB), and 3) controlling upsetting thoughts (CUT).

**Methods:**

Baseline data among a sample of caregivers (*n* = 144) were examined. An ANCOVA was conducted with SES (race, education level, household income, employment, and insurance status) as independent variables and scores from the three domains measured by the Revised Scale for Caregiving Self‐Efficacy, as dependent variables.

**Results:**

There were no significant differences in self‐efficacy related to obtaining respite and RDPB between SES groups. There were significant differences in the CUT domain scores between household income (F=4.615, *p* < .05) and insurance status groups (F=3.432, *p* < .01). Pairwise comparisons revealed that individuals with private health insurance or Medicaid reported higher self‐efficacy in RDPB when compared to individuals covered by Medicare and another insurance type (e.g. Medicare Advantage) (*p* < .01; *p* < .05). Individuals with graduate degrees had significantly higher RDPB scores when compared to those with trade school/some college/associates degrees (*p* < .05). In the CUT domain, full time employees reported higher self‐efficacy than those who were unemployed (*p* < .05), and Medicaid recipients scored significantly higher than individuals with private insurance (*p* < .05), Medicare+ (*p* < .05) or other insurance (*p* < .01).

**Conclusion:**

It is important to consider SES when designing interventions for ADRD caregivers. Our results indicate that insurance status, educational level, and employment status may be related to self‐efficacy among ADRD caregivers, specifically regarding responses to disruptive behaviors and controlling upsetting thoughts. For example, those with private insurance and Medicaid may have access to more resources that teach them to cope and manage disruptive behaviors. Education level may also be related to factors that can increase self‐efficacy such as understanding ADRD symptoms and how to manage them. Future studies should examine mediating factors and examine differences among a larger sample.

